# Editorial: Explicit and implicit emotion processing: neural basis, perceptual and cognitive mechanisms, volume II

**DOI:** 10.3389/fpsyg.2024.1411947

**Published:** 2024-05-09

**Authors:** Noemi Mazzoni, Alessia Celeghin, Giulia Mattavelli

**Affiliations:** ^1^Department of Psychology “Renzo Canestrari”, University of Bologna, Cesena, Italy; ^2^Department of Psychology, University of Turin, Turin, Italy; ^3^ICoN Center, Scuola Universitaria Superiore IUSS, Pavia, Italy; ^4^Istituti Clinici Scientifici Maugeri IRCCS, Cognitive Neuroscience Laboratory of Pavia Institute, Pavia, Italy

**Keywords:** implicit emotion, explicit emotion, social cognition, psychiatric disorder, virtual reality, NIBS

The investigation of emotion processing is a compelling research area that has experienced significant growth over the past decades. Yet, the relationship between explicit and implicit processing of emotional and non-emotional stimuli remains unclear, especially in pediatric and clinical populations. Additionally, the role of gender differences remains poorly elucidated. Nevertheless, despite decades of research in the field consistently highlighting the intricate interplay between emotions and cognition, the reciprocal influence between different cognitive domains and the impact of endogenous and exogenous factors on affective neural and behavioral responses remain underexplored.

With the aim of advancing our comprehension of emotion processing and encoding, this Research Topic seeks to gather applied studies employing innovative methodologies. Indeed, if the ultimate goal of emotion processing research is to understand human behavior across development, whether typical or atypical, a broader and more inclusive perspective is imperative.

Oldrati et al. addressed some of these criticalities by investigating the interplay between emotion processing and attention in children and adolescents. Administering Flanker tasks and a same-or-different judgment paradigm, the authors tested whether the processing of emotional and sexual (i.e., not emotional) information from faces and bodies was modulated by top-down and bottom-up attentional mechanisms. Notably, children and adolescents were able to filter out the emotional features when the task required space-based filtering (Flanker task), while they failed to do so when the task required a feature-based filtering mechanism (same-or-different judgment task). Indeed, in the Flanker tasks, only the sex features influenced the allocation of attention either when task-relevant (top-down) or when task-irrelevant (bottom-up), while in the same-or-different judgment task, both the emotional and sexual task-irrelevant cues intruded on the processing of the targets. These results extend previous findings from the adult to pediatric population, supporting the mutual influence between emotion processing and attentional mechanisms (either top-down or bottom-up) in developmental age. Nevertheless, the findings highlight that the influence of emotions on cognitive processes is highly susceptible to methodological choices.

The relevance of methodological aspects in relation to clinical populations of interest also emerges from the studies by Vaioli et al. and Telesca et al.. The former assessed the recognition of fearful and angry facial expressions in women with anorexia nervosa (AN) using an implicit recognition task. Interestingly, the paradigm aimed to elicit the redundant target effect (Miniussi et al., 1998; Tamietto et al., 2006; Georgy et al., 2016), which posits that the detection and recognition of a visual target is facilitated by simultaneous events and hindered by competing ones. The results showed altered behavioral performance in AN participants in processing fearful, but not angry, facial expressions, which was associated with the level of alexithymia. This suggests that fear processing may be influenced by individual experiences and contextual cues, such as personal goals and internal states, shaping the subjective relevance and subsequent appraisal of emotional stimuli (Roseman and Smith, 2001).

The study by Telesca et al. investigated social cognition abilities, including facial emotion recognition, in patients with chronic pain classified as primary or secondary pain according to the latest disease classification (Nicholas et al., 2019). This is a novelty, as no previous evidence exists about possible differences in emotion processing between the two conditions. Social cognition impairments were detected only in patients with chronic secondary pain (i.e., pain was a consequence of another medical condition) and correlated with global cognitive functioning, underscoring the intertwined nature of emotion recognition with cognitive abilities.

These findings have crucial implications, as they inform clinicians about the pivotal interplay between psychological and affective factors in pathologies in which the processing of emotions is not the focus of the symptomatology but is nevertheless central to the quality of life and treatments (Lavender et al., 2015; Koechlin et al., 2018).

The importance of addressing emotional impairment to improve social functioning has also been reported in schizophrenia (Gorrino et al., 2024). Vergallitto et al. integrated psychological interventions with non-invasive brain stimulation (NiBS) over the dorsolateral prefrontal cortex to boost the neuroplastic changes induced by the training. NiBS has received increasing attention as a treatment for psychiatric disorders (Hyde et al., 2022) as it can rebalance altered neural activity, inducing long-lasting effects. It is well-established that NiBS effects are state-dependent (Silvanto et al., 2008; Pisoni et al., 2018; Sack et al., 2023). Thus, combining NiBS with personalized psychological training is posited to engender a synergistic effect between brain stimulation and social-cognitive intervention. This hypothesis has been explored in a pilot randomized control trial with schizophrenic patients (Vergallito et al.). Despite the absence of statistically significant findings, the methodology presented in this study is innovative and promising and offers new insight into the potential integration of NiBS with psychological intervention to enhance social cognition abilities.

Finally, the impact of emotion processing in daily life activities has been investigated by Mohamed Aly et al. exploring the effects of exposure to emotional faces, bodies, or landscapes on wayfinding behavior. In a series of experiments using virtual reality, they reported that negative emotions influence spatial navigation differently in female and male individuals, highlighting the importance of gender differences in emotion processing, not only in terms of different mechanisms recruited by male and female individuals (Proverbio et al., 2006), but also in terms of different impacts on daily activities, such as spatial navigation (Castillo et al., 2021).

In sum, the studies included in this Research Topic well represent the interdisciplinary nature of emotion research. Innovative methodologies have been presented, including studying emotion processing in a virtual reality environment (Mohamed Aly et al.) and combining brain stimulation with cognitive intervention (Vergallito et al.). New evidence has emerged, showing the interplay between emotion encoding and cognitive processes, such as attention (Oldrati et al.), spatial navigation (Mohamed Aly et al.), and global cognitive functioning (Telesca et al.). This is crucial in pediatric and clinical populations, where these variables can be highly heterogeneous and have a dramatic impact on the effectiveness of training and intervention and, in turn, on the social and relational aspects of daily life.

## Author contributions

NM: Writing—original draft, Writing—review & editing. AC: Writing—original draft, Writing—review & editing. GM: Writing—original draft, Writing—review & editing.
